# Ethical concerns toward medical artificial intelligence and acceptance intentions: a structural equation modeling analysis of the risk perception–trust pathway

**DOI:** 10.3389/fpubh.2026.1858239

**Published:** 2026-08-25

**Authors:** Shengli Gu, Jie Zhang

**Affiliations:** Division of Medicine, Nantong University Xinglin College, Nantong, China

**Keywords:** acceptance, ethical concern, health policy, medical artificial intelligence, risk perception

## Abstract

**Objective:**

With the deep integration of artificial intelligence (AI) into medical imaging, clinical decision-making, and health management, ethical concerns regarding medical AI among surveyed individuals have become increasingly prominent. This study examines the hierarchical structure of ethical concerns among surveyed participants and their associations with perceived risk, trust, attitude, and acceptance of medical AI.

**Methods:**

A questionnaire survey was conducted with 697 valid responses. SPSS and AMOS were used for reliability and validity assessment, confirmatory factor analysis, structural equation modeling, bootstrap analysis of indirect pathways, and robustness checks.

**Results:**

The results support a hierarchical multidimensional structure of ethical concern, with six first-order dimensions—privacy and data protection, responsibility and accountability, fairness and accessibility, safety and reliability, human–machine collaboration and humanistic care, and technical interpretability and transparency—collectively representing a higher-order ethical concern construct. All six dimensions are significantly associated with higher overall perceived risk of medical AI. Among them, human–machine collaboration and humanistic care (*β* = 0.238), fairness and accessibility (*β* = 0.196), and technical interpretability and transparency (*β* = 0.187) show relatively stronger associations. Overall perceived risk is negatively associated with overall trust (*β* = −0.240), while overall trust is positively associated with overall attitude (*β* = 0.608) and overall acceptance (*β* = 0.395). Overall attitude is positively associated with overall acceptance (*β* = 0.397). Personal trust propensity is positively associated with overall trust (*β* = 0.555). Bootstrap analysis indicates that overall perceived risk shows an indirect statistical association with overall acceptance involving overall trust within the structural model. Sensitivity analysis incorporating demographic control variables (gender, age, education level, and prior experience with medical AI) indicates that the structural relationships remain consistent.

**Conclusion:**

Ethical concerns among surveyed participants constitute a hierarchical construct that is systematically associated with perceived risk of medical AI. Perceived risk, trust, attitude, and acceptance are interconnected through multiple statistical pathways, highlighting the complexity of medical AI evaluation processes among surveyed participants.

## 1 Introduction

With the promotion of policy documents such as the Opinions on Further Implementing the “Artificial Intelligence+” Initiative issued by the State Council of China, the application of artificial intelligence (AI) in the medical field has entered a stage of rapid development (1). Medical AI has gradually become an important tool for optimizing the allocation of medical resources and improving diagnostic and treatment efficiency. However, ethical risks such as privacy and data protection, fairness and accessibility, responsibility and accountability, safety and reliability, human–machine collaboration and humanistic care, and technical interpretability and transparency have also become more prominent in real-world applications.

Technology acceptance research has extensively relied on the Technology Acceptance Model (TAM) and the Unified Theory of Acceptance and Use of Technology (UTAUT) to explain user adoption of emerging technologies (2, 3). These frameworks have been widely applied in information systems, healthcare, and artificial intelligence adoption research, and have been progressively extended to incorporate additional psychological mechanisms to better explain complex adoption behaviors. In particular, empirical studies across these domains have increasingly incorporated perceived risk and trust as key explanatory constructs, reflecting a broader effort to enhance the explanatory power of traditional acceptance models in high-uncertainty environments (4–6).

Across information systems, automation, and healthcare AI research, empirical studies have consistently examined the relationships among perceived risk, trust, and acceptance (7, 8). Perceived risk is generally understood as a multidimensional psychological construct involving individuals’ cognitive evaluations and affective responses toward uncertain outcomes (9). While these studies generally report meaningful associations among these constructs, the strength and structural patterns of these relationships vary across contexts and application domains, particularly in safety-critical settings such as healthcare (10). Accordingly, the risk–trust–acceptance relationship is better understood as a recurring empirical pattern rather than a fully unified theoretical mechanism. Within this broader literature stream, existing research has not yet systematically examined how different ethical concerns jointly shape perceived risk and subsequently influence trust and acceptance in medical AI contexts.

Ethical concern, rooted in moral sensitivity theory, refers to individuals’ capacity to recognize and evaluate ethically relevant issues in specific contexts (11). Recent AI ethics frameworks further emphasize privacy and data protection, fairness and accessibility, responsibility and accountability, safety and reliability, human–machine collaboration and humanistic care, and technical interpretability and transparency as core governance principles (12). Although these ethical domains originate from a shared concern regarding responsible medical AI governance, they represent theoretically distinct aspects of ethical evaluation. Therefore, ethical concern is conceptualized in this study as a hierarchical multidimensional construct: the six dimensions represent first-order ethical domains, while their shared variance reflects an overarching higher-order ethical concern toward medical AI. However, existing studies have not systematically decomposed these ethical concerns into distinct dimensions and linked them to risk–trust–acceptance mechanisms in individual evaluations of medical AI.

To address this gap, this study proposes the following research questions:

(1) What are the constituent dimensions of ethical concern perceived by surveyed participants in the context of medical AI applications?
(2) How are different ethical dimensions associated with overall perceived risk, and how is this subsequently related to overall trust and overall acceptance of medical AI?

Building on these questions, this study develops a theoretical model integrating six dimensions of ethical concern, overall perceived risk, overall trust, and overall acceptance. A key contribution of this study is the development of a hierarchical multidimensional ethical concern framework within the risk–trust–acceptance model (2, 3). Rather than treating ethical concern as a homogeneous concept, this study identifies six theoretically distinct ethical domains while recognizing their shared foundation as an overarching ethical concern toward medical AI.

This study contributes to the literature in two ways. First, it extends prior technology acceptance research by incorporating a hierarchical multidimensional ethical concern structure into the existing risk–trust–acceptance framework. Second, it provides a more fine-grained explanation of how specific ethical dimensions are associated with acceptance of medical AI among surveyed participants through overall perceived risk and overall trust.

## 2 Research hypotheses and theoretical model

With the rapid expansion of artificial intelligence in medical imaging analysis, clinical decision support, and health management, ethical and governance issues have become increasingly central in both research and practice. A substantial body of interdisciplinary research in AI ethics, healthcare AI, and information systems has highlighted key ethical concerns in AI deployment, including privacy, accountability, fairness, safety, interpretability, and human-centered considerations (12, 13).

Within this research landscape, the World Health Organization (WHO) has provided formal guidance on the Ethics and Governance of Artificial Intelligence for Health, outlining a set of core normative principles for responsible AI development and deployment in healthcare contexts (14). These principles are widely consistent with those identified in empirical AI ethics and governance literature, and have further informed subsequent operational frameworks for healthcare AI governance (15).

Building on value-sensitive design theory, this study conceptualizes ethical concern as a hierarchical multidimensional construct consisting of six first-order dimensions: privacy and data protection, responsibility and accountability, fairness and accessibility, safety and reliability, human–machine collaboration and humanistic care, and technical interpretability and transparency. These dimensions represent distinct ethical domains while jointly reflecting an overarching ethical concern toward medical AI.

Prior research across information systems, healthcare AI, and human–AI interaction has examined how ethical considerations shape users’ cognitive and evaluative responses to intelligent systems. Rather than functioning as isolated determinants, ethical concerns are generally embedded within broader assessment processes through which users evaluate uncertainty, reliability, and potential risks in AI-enabled environments.

Within the broader technology acceptance and automation literature, perceived risk has been widely established as a key explanatory construct. From a psychological perspective, risk perception reflects individuals’ subjective evaluation of uncertain outcomes and potential consequences rather than objective risk alone (9). Trust is recognized as a central mechanism linking cognitive evaluation to attitudinal and behavioral responses toward AI systems (16). Although these relationships have been consistently reported, their integration remains context-dependent, particularly in high-stakes healthcare applications (17).

**Figure 1:**
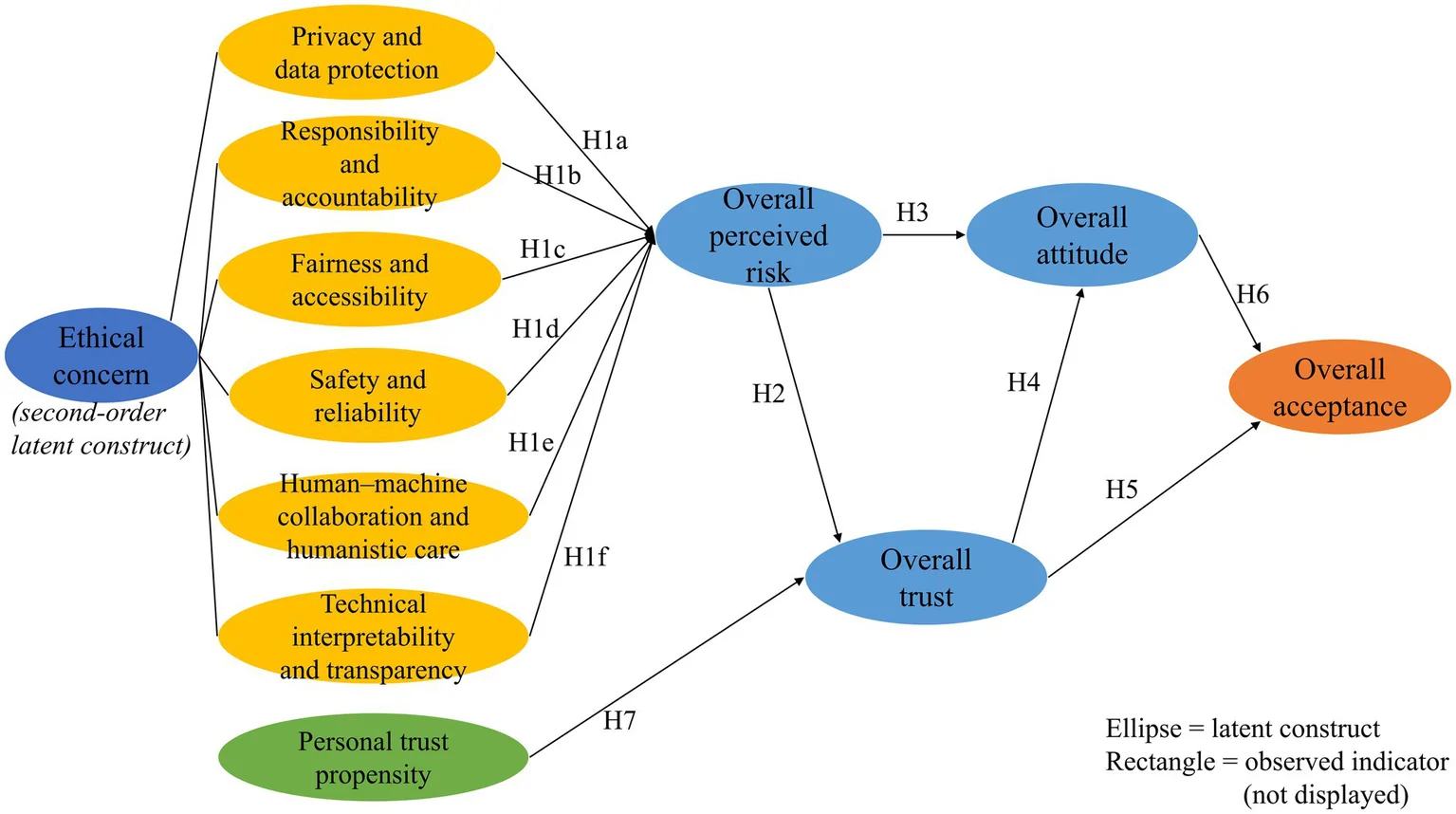
Hypothesized hierarchical model of ethical concern, perceived risk, trust, attitude, and acceptance. The second-order construct “ethical concern” is represented by six first-order dimensions. Observed questionnaire items are omitted for clarity and are reported in Supplementary Table S1.

Accordingly, this study positions perceived risk, trust, and attitude as sequential psychological mechanisms linking ethical concern to acceptance outcomes. Individual trust propensity is further incorporated as a stable dispositional factor influencing initial trust formation under uncertainty. The hypothesized model is shown in Figure 1.

It should be noted that all six ethical concern dimensions are operationalized as the degree of public worry about each ethical issue; whereas overall perceived risk represents a global integrated evaluation of uncertainty and potential harm.

Medical data are inherently sensitive. In clinical AI research, privacy breaches have been shown to heighten perceived risk in AI-enabled healthcare settings (4); any leakage, misuse, or unauthorized access during data collection, storage, transmission, or use is likely to intensify public concerns about overall AI risks. Accordingly:

*H1a*: Concern about privacy and data protection is positively associated with overall perceived risk.

Medical AI introduces a fundamental question: who is liable when things go wrong? When responsibility is diffuse and accountability mechanisms are absent, uncertainty about legal and institutional recourse amplifies public risk perception (18). A clear accountability structure is therefore central to how the public evaluates the safety of AI in healthcare. Accordingly:

*H1b*: Concern about responsibility and accountability is positively associated with overall perceived risk.

If medical AI systematically benefits some groups while disadvantaging others, its legitimacy is called into question (19). The perception that a technology may reinforce existing inequalities or exclude vulnerable populations is, in itself, a source of perceived risk. Accordingly:

*H1c*: Concern about fairness and accessibility is significantly positively associated with overall perceived risk.

At its core, the public’s willingness to trust medical AI depends on whether the technology can be depended upon in moments of clinical uncertainty (10). When system failures, misdiagnoses, or loss of control are perceived as plausible outcomes, the overall risk appraisal rises correspondingly. Hence:

*H1d*: Concern about safety and reliability is significantly positively associated with overall perceived risk.

A common worry about medical AI is not just whether it works, but what it displaces: physician judgment, patient–clinician communication, and the human touch in care delivery (7). The more people perceive AI as a threat to the relational and empathetic dimensions of medicine, the more they tend to view it as risky. Therefore:

*H1e*: Concern about human–machine collaboration and humanistic care is significantly positively associated with overall perceived risk.

Medicine has long demanded that decisions be justified and traceable. When AI systems fail to provide clear reasoning or make their internal logic accessible, the resulting opacity becomes a direct source of perceived risk (20). Accordingly:

*H1f*: Concern about technical interpretability and transparency is significantly positively associated with overall perceived risk.

Trust requires a belief that the other party is competent and dependable. When risk perception is high, this belief becomes harder to sustain (6, 21). In the medical AI context, heightened uncertainty about system performance and potential harm undermines the public’s willingness to trust. Therefore:

*H2*: Overall perceived risk is significantly negatively associated with overall trust.

Public attitudes toward medical AI are shaped in part by how individuals evaluate the potential risks associated with the technology (5). Although higher perceived risk may increase concerns and uncertainty, it may also stimulate more deliberate evaluations of potential benefits and limitations. Therefore, the relationship between perceived risk and attitude may depend on how individuals balance potential risks against expected benefits. Based on the traditional risk perception perspective, the following hypothesis is proposed:

*H3*: Overall perceived risk is significantly negatively associated with overall attitude.

Trust functions as a psychological bridge: when people trust a technology, they are more inclined to view it favorably (6). In healthcare AI, trust reduces the emotional distance between users and the system, making positive attitudes more likely. Thus:

*H4*: Overall trust is significantly positively associated with overall attitude.

Trust is a necessary condition for adoption in high-stakes settings (7). People are unlikely to accept a technology they do not trust, particularly when it affects their health. Higher trust therefore predicts greater acceptance intentions. Accordingly:

*H5*: Overall trust is significantly positively associated with overall acceptance.

Attitudes capture an individual’s overall evaluation of a technology; these evaluations tend to guide behavioral intentions (22). A positive attitude toward medical AI is therefore a reliable predictor of acceptance. Thus:

*H6*: Overall attitude is significantly positively associated with overall acceptance.

Not everyone starts from the same baseline. Some individuals are simply more disposed to trust new technologies, and this disposition shapes their initial response to medical AI (17, 23). Those with higher general trust propensity tend to form stronger trust even before accumulating direct experience. Accordingly:

*H7*: Individual trust propensity is significantly positively associated with overall trust.

From the perspective of the psychological processes underlying public acceptance of medical AI, overall perceived risk may be statistically associated with overall acceptance through indirect pathways involving overall trust. Specifically, perceived risk may be related to individuals’ evaluations of the reliability and trustworthiness of medical AI systems, which may subsequently be reflected in their acceptance intentions. Therefore, based on the indirect pathway specified in the structural model, the following hypothesis is proposed:

Overall trust may represent an indirect statistical pathway associated with the relationship between overall perceived risk and overall acceptance.

## 3 Research methods

### 3.1 Questionnaire design

The ethical concern construct was modeled hierarchically. The six ethical dimensions were operationalized as first-order constructs because they represent different ethical domains, whereas their common conceptual foundation was examined through a second-order factor structure.

The questionnaire adopts a structured design and mainly consists of three parts: (1) Basic information: including the respondent’s gender, age, education, occupational background, and experience with medical AI. (2) Ethical perception dimensions of medical AI: assessing the respondent’s subjective feelings about the ethical performance of medical AI in practical application, scored using a 7-point Likert scale (1 = strongly disagree, 7 = strongly agree). (3) Public attitude toward medical AI: including trust, acceptance, and overall attitude.

Based on mature scales, this study adjusted the expression of existing items to fit the core variables of this study. All constructs were measured using a 7-point Likert scale. We acknowledge that some items (e.g., “I support establishing more comprehensive AI medical laws”) reflect normative policy preferences arising from ethical concerns rather than direct worry. These items are retained because they capture the behavioral-intention component of ethical concern. The operational definitions of each core construct are shown in Table 1.

**Table 1:** Operational definitions of variables.

Variable	Definition	References
Personal trust propensity	An individual’s general tendency to initially trust medical AI technologies in the absence of detailed information about specific applications.	Hoff KA (17)
Concern about privacy and data protection	The degree to which an individual worries that the entire process of collecting, storing, processing, and sharing personal health information by a medical AI system may infringe on their information autonomy and security.	Cao, Meng (22)
Concern about responsibility and accountability	The degree to which an individual worries that it is difficult to clearly and fairly determine and hold responsible parties when a medical AI system causes errors or damages.	Shi Yun Ng (26)
Concern about fairness and accessibility	The degree to which an individual worries that medical AI may lead to discriminatory results or exacerbate unequal access to medical services for different groups due to algorithmic bias or uneven resource allocation.	Cao, Meng (22)
Concern about safety and reliability	The degree to which an individual worries that medical AI systems may fail due to technical errors, algorithmic malfunction, or system instability, potentially causing harm to patients.	Cao, Meng (22)
Concern about human–machine collaboration and humanistic care	The degree to which an individual worries that the application of medical AI may weaken necessary interpersonal interaction and emotional support between doctors and patients and lead to inappropriate replacement of doctors’ roles.	Weir IB (27)
Concern about technical interpretability and transparency	The degree to which an individual worries about the uncertainty caused by the opaque and incomprehensible decision-making logic of medical AI (i.e., the “black box” problem), which hinders understanding, trust, and responsibility tracing.	Pierce RL (20)
Overall perceived risk	The subjective judgment of an individual regarding the overall negative consequences and uncertainty that may arise from using medical AI after comprehensively considering various ethical concerns.	Townsend BA (10)
Overall trust	The strength of an individual’s belief that medical AI is reliable, honest, and focused on patient well-being in specific scenarios after considering the context and risks.	Shao MM (6)
Overall acceptance	The specific behavioral intention of an individual to use or accept AI-assisted diagnosis and treatment in actual medical services in the future.	Davis, Venkatesh (2, 3)
Overall attitude	The overall positive or negative emotional and cognitive evaluation of an individual regarding the development and application of AI technology in the medical field.	Cao, Meng (22)

### 3.2 Sample collection

A pre-survey was conducted before formal data collection to test the rationality of the scale structure. The pre-survey sample included 94 respondents. Cronbach’s *α* coefficients showed that the reliability of each construct was higher than 0.80, indicating good internal consistency of the scale.

The formal survey employed a multi-channel recruitment strategy. First, an initial seed sample was recruited through the online survey platform Wenjuanxing disseminated via social media (WeChat) through snowball referral. Second, offline paper questionnaires were distributed in community health centers in three Chinese cities (Beijing, Shanghai, and Nanjing), with approximately 50 valid responses collected. No monetary incentives were offered. We acknowledge that the snowball component represents a non-probability sampling approach, and the combined sample should not be interpreted as strictly representative of the general population. All respondents were required to be able to read and complete the questionnaire independently, and only fully completed questionnaires were included in the final dataset.

Ethical approval for this study was obtained from the Ethics Committee of Nantong University (Approval No.(202) of the Ethics Review Committee of Nantong University). Informed consent was obtained from all participants prior to participation. For the online survey conducted via Wenjuanxing, a consent information page was presented at the beginning of the questionnaire, providing a full description of the study purpose, procedures, and data protection measures. Participants could only proceed to the questionnaire after actively selecting “I agree,” thereby constituting informed electronic consent. For the offline paper-based survey, participants were first provided with a written informed consent form, which they read and signed before completing the questionnaire. The study was conducted in accordance with the Declaration of Helsinki.

## 4 Data analysis and results

### 4.1 Sample characteristics

A total of 702 questionnaires were collected in this study. After data quality screening (excluding straight-lining responses and implausibly short completion times) and excluding incomplete questionnaires, 697 valid samples were finally retained (697 of 702, retention rate = 99.29%). Among the respondents, males accounted for 50.36% and females 49.64%. The largest age group was 18–25 years (26.26%), followed by relatively balanced distributions across other age categories. In terms of education level, 41.18% of respondents held a bachelor’s degree, and 56.96% held a bachelor’s degree or above. In terms of technical experience, about 41% reported having direct contact with medical AI applications, including AI-assisted diagnostic tools, AI-enabled health management applications, or AI-based medical consultation platforms. The details are shown in Table 2.

**Table 2:** Demographic characteristics.

Name	Options	Frequency	Percentage (%)
Gender	Male	351	50.36
	Female	346	49.64
Age group	18–25	183	26.26
	26–35	168	24.10
	36–45	163	23.39
	46–55	118	16.93
	56 and above	65	9.33
Highest education	Bachelor	287	41.18
	Junior college	194	27.83
	High school and below	106	15.21
	Master	86	12.34
	Doctor	24	3.44
Used medical AI	No	409	58.68
	Yes	288	41.32

### 4.2 Descriptive statistics and reliability and validity analysis

**Figure 2:**
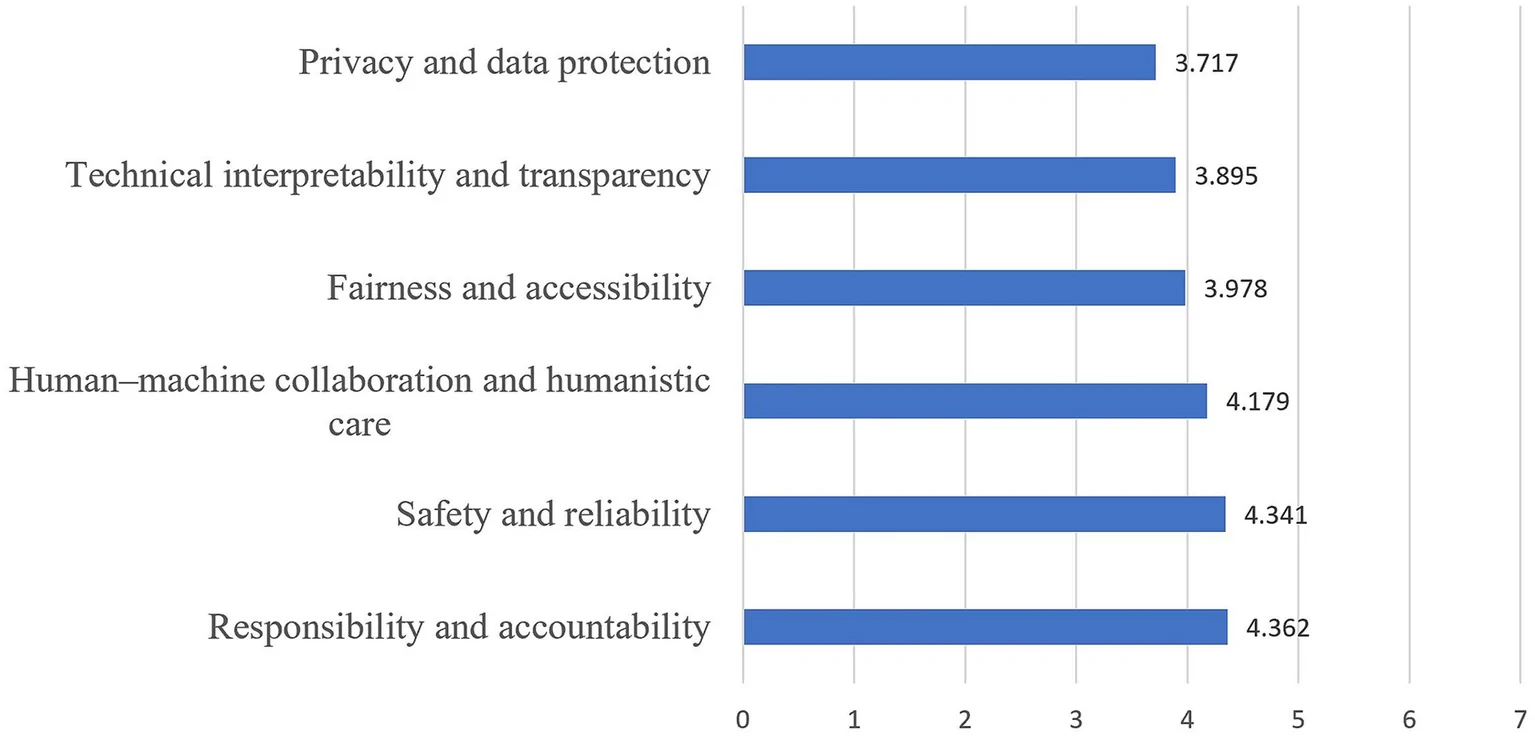
Mean scores of ethical concern dimensions. Note: Scores were measured on a 7-point Likert scale, with higher values indicating greater concern regarding the corresponding ethical issues.

Overall, the descriptive statistics indicated that the distributions of the constructs were generally acceptable (see Table 3). To provide an intuitive comparison of the six ethical concern dimensions, the mean scores of these dimensions are further visualized in Figure 2. Although some deviations from strict normality were observed for certain constructs (e.g., overall trust: skewness = −1.412, kurtosis = 3.146), these deviations were considered acceptable given the large sample size (*N* = 697). Accordingly, maximum likelihood (ML) estimation was retained for SEM analysis, and bootstrap procedures with 5,000 resamples were conducted to examine the stability of parameter estimates.

**Table 3:** Mean and standard deviation of main variables.

Variable name	Mean ± standard deviation	Kurtosis	Skewness
Overall acceptance	5.136 ± 1.193	0.524	−0.731
Overall trust	5.73 ± 1.049	3.146	−1.412
Overall attitude	5.171 ± 1.148	0.826	−0.764
Overall perceived risk	3.999 ± 1.413	−0.592	0.238
Technical interpretability and transparency	3.895 ± 1.586	−0.779	0.24
Human–machine collaboration and humanistic care	4.179 ± 1.401	−0.63	0.142
Safety and reliability	4.341 ± 1.463	−0.68	−0.108
Fairness and accessibility	3.978 ± 1.465	−0.685	0.256
Responsibility and accountability	4.362 ± 1.464	−0.751	0.021
Privacy and data protection	3.717 ± 1.501	−0.693	0.299
Personal trust propensity	4.836 ± 1.184	0.251	−0.526

Given that all data were collected through self-reported questionnaires at a single time point, potential common method variance (CMV) was assessed. First, Harman’s single-factor test indicated that the first factor accounted for 38.77% of the total variance, which was slightly below the commonly used 40% reference threshold. However, considering the limited sensitivity of this approach, the Harman test was treated only as preliminary evidence regarding potential CMV rather than definitive evidence of its absence.

To further evaluate potential CMV, a Common Latent Factor (CLF) approach was conducted in AMOS. A latent method factor was introduced into the measurement model, allowing all observed indicators to load simultaneously on their theoretical constructs and the common latent factor. The comparison between the baseline CFA model and the CFA + CLF model is presented in Table 4.

**Table 4:** Comparison of CFA and CFA + CLF model fit indices.

Fit index	CFA model	CLF model	Δ (change)
*χ*^2^/*df*	1.484	1.480	0.004
GFI	0.919	0.919	0.000
CFI	0.986	0.986	0.000
TLI	0.984	0.984	0.000
NFI	0.957	0.957	0.000
RMSEA	0.026	0.026	0.000
RMR	0.066	0.065	0.001

The results showed that the inclusion of the common latent factor resulted in negligible changes in global model fit indices (ΔCFI = 0.000, ΔRMSEA = 0.000). Although all indicators showed significant loadings on the CLF, the magnitude of these loadings (0.336–0.504) remained lower than the corresponding theoretical construct loadings. Detailed CLF loading results are reported in Supplementary Table S3. These findings suggest that a certain degree of common method influence may exist; however, the substantially stronger theoretical construct loadings and negligible changes in model fit indicate that CMV is unlikely to account for the majority of variance in the observed relationships. Nevertheless, given the cross-sectional self-reported design, the potential influence of CMV cannot be completely excluded and is acknowledged as a limitation.

Reliability and convergent validity were assessed using Cronbach’s *α*, composite reliability (CR), average variance extracted (AVE), and standardized factor loadings (see Table 5).

**Table 5:** Measurement model reliability and convergent validity.

Variable	Standardized loading coefficient	AVE value	CR value	Cronbach’s α coefficient
Personal trust propensity (adapted from Mayer and Davis, 1999; based on Rotter, 1967 (28, 29))	0.782–0.855	0.656	0.884	0.884
Privacy and data protection	0.857–0.909	0.786	0.936	0.935
Responsibility and accountability	0.851–0.910	0.797	0.940	0.940
Fairness and accessibility	0.846–0.890	0.764	0.928	0.928
Safety and reliability	0.870–0.909	0.784	0.936	0.935
Human–machine collaboration and humanistic care	0.849–0.885	0.759	0.926	0.926
Technical interpretability and transparency	0.904–0.908	0.821	0.948	0.948
Overall perceived risk (developed based on the AI Risk–Benefit Scale (AIRBS) and AI risk literature (30))	0.877–0.887	0.779	0.946	0.946
Overall attitude (adapted from the artificial intelligence attitude scale-4 (AIAS-4); based on UTAUT framework (3, 31))	0.840–0.858	0.721	0.912	0.912
Overall trust (adapted from McKnight et al., 2002; based on Mayer et al. trust framework (32, 33))	0.838–0.869	0.730	0.915	0.915
Overall acceptance (adapted from Davis, 1989; Venkatesh et al., 2003 (2, 3))	0.838–0.892	0.746	0.922	0.922

AIRBS refers to the AI Risk–Benefit Scale proposed by Kerstan et al. (30), and AIAS-4 refers to the artificial intelligence attitude scale-4 developed by Grassini (31).

The results indicate strong internal consistency, with Cronbach’s α values for all constructs exceeding 0.88. CR values ranged from 0.884 to 0.948, exceeding the recommended threshold of 0.70. All AVE values ranged from 0.656 to 0.821, above the 0.50 criterion. Standardized factor loadings were all above 0.78, supporting adequate convergent validity. To address potential construct heterogeneity between normative-policy-oriented and worry-based items, we clarify that these items capture two complementary cognitive–evaluative processes underlying responsibility and accountability perceptions in medical AI. Normative items reflect evaluative judgments regarding the adequacy of governance and regulatory frameworks, whereas worry-based items reflect perceived uncertainty arising from insufficient accountability structures. Importantly, these two types of items converge at the latent level because both reflect individuals’ overall evaluative appraisal of accountability robustness rather than distinct conceptual domains. Empirically, all items exhibit consistently high factor loadings (0.85–0.91) on a single latent construct, with no evidence of cross-factor divergence, indicating a stable unidimensional measurement structure. This convergence suggests that normative and affective components operate as different expressions of a unified underlying evaluative orientation toward accountability in medical AI.

Confirmatory factor analysis (CFA) demonstrated good model fit: *χ*^2^/*df* = 1.484, GFI = 0.919, CFI = 0.986, TLI = 0.984, NFI = 0.957, RMSEA = 0.026, indicating that the measurement model fits the data well (see Table 6).

**Table 6:** Model fit indicators.

Common indicator	*X* ^2^	*df*	P	*χ*^2^/*df*	GFI	RMSEA	RMR	CFI	NFI	NNFI
Judgment standard	—	—	>0.05	<3	>0.9	<0.10	<0.05	>0.9	>0.9	>0.9
Value	1320.855	890	0	1.484	0.919	0.026	0.066	0.986	0.957	0.984

Given that the six ethical concern dimensions showed relatively high correlations due to their shared conceptual foundation, a second-order confirmatory factor analysis (CFA) was further conducted to examine whether these dimensions could be represented by an overarching ethical concern construct. In this model, privacy and data protection, responsibility and accountability, fairness and accessibility, safety and reliability, human–machine collaboration and humanistic care, and technical interpretability and transparency were specified as first-order constructs loading onto a higher-order ethical concern factor.

The second-order CFA demonstrated excellent model fit (*χ*^2^/*df* = 1.576, GFI = 0.952, CFI = 0.992, TLI = 0.991, NFI = 0.978, RMSEA = 0.029), indicating that the hierarchical structure provided an adequate representation of ethical concern. Furthermore, all six first-order dimensions showed significant and strong standardized loadings on the higher-order ethical concern construct, ranging from 0.794 to 0.873 (*p* < 0.001). Specifically, technical interpretability and transparency showed the strongest loading (*λ* = 0.873), followed by fairness and accessibility (*λ* = 0.843), while safety and reliability showed the lowest loading (*λ* = 0.794). These results support the conceptualization of ethical concern as a hierarchical multidimensional construct, in which different ethical domains represent distinct aspects while collectively reflecting a broader underlying ethical concern toward medical AI (see Table 7).

**Table 7:** Second-order factor loadings of ethical concern.

First-order dimensions	Standardized loading (*λ*)	C.R.	*p*-value
Privacy and data protection	0.811	-	-
Responsibility and accountability	0.815	20.00	<0.001
Fairness and accessibility	0.843	20.241	<0.001
Safety and reliability	0.794	19.061	<0.001
Human-machine collaboration and humanistic care	0.818	19.640	<0.001
Technical interpretability and transparency	0.873	21.567	<0.001

Ethical concern was modeled as a second-order latent construct. Standardized loadings represent the relationships between the higher-order ethical concern construct and its six first-order dimensions.

Discriminant validity was assessed using both the Fornell–Larcker criterion and the Heterotrait–Monotrait (HTMT) ratio.

The Fornell–Larcker results show that the square roots of AVE for each construct are higher than the corresponding inter-construct correlations, indicating acceptable discriminant validity (see Table 8).

**Table 8:** Discriminant validity analysis.

Variable	Personal trust propensity	Privacy and data protection	Responsibility and accountability	Fairness and accessibility	Safety and reliability	Human–machine collaboration and humanistic care	Technical interpretability and transparency	Overall perceived risk	Overall trust	Overall acceptance	Overall attitude
Personal trust propensity	0.81										
Privacy and data protection	0.169	0.887									
Responsibility and accountability	0.13	0.624	0.893								
Fairness and accessibility	0.109	0.634	0.625	0.874							
Safety and reliability	0.076	0.604	0.611	0.628	0.885						
Human–machine collaboration and humanistic care	0.099	0.608	0.639	0.634	0.616	0.871					
Technical interpretability and transparency	0.097	0.658	0.668	0.708	0.642	0.667	0.906				
Overall perceived risk	0.112	0.701	0.711	0.728	0.669	0.729	0.748	0.883			
Overall trust	0.45	0.041	−0.148	−0.116	−0.121	−0.122	−0.148	−0.162	0.854		
Overall acceptance	0.422	0.244	0.027	0.049	0.015	0.001	0.052	0.048	0.573	0.864	
Overall attitude	0.496	0.07	0.038	0.073	0.011	0.007	0.051	−0.008	0.525	0.578	0.849

To further ensure robustness, the HTMT ratio was calculated for all latent constructs. HTMT is considered a more stringent criterion for assessing discriminant validity in structural equation modeling. The results indicate that all HTMT values are below the recommended threshold of 0.85 (and also below the more conservative threshold of 0.90), supporting acceptable discriminant validity among the constructs, while acknowledging that the six ethical concern dimensions share a common conceptual foundation.

Although the ethical concern dimensions exhibit relatively high correlations due to their shared ethical foundation, the HTMT results indicate that they maintain sufficient empirical distinction. Combined with the second-order CFA results, these findings suggest that the six dimensions are neither redundant nor entirely independent; rather, they represent distinct manifestations of a broader ethical concern construct (see Table 9).

**Table 9:** Heterotrait-monotrait ratio (HTMT) - Matrix.

Variable	Fairness and accessibility	Human–machine collaboration and humanistic care	Overall acceptance	Overall attitude	Overall perceived risk	Overall trust	Personal trust propensity	Privacy and data protection	Responsibility and accountability	Safety and reliability	Technical interpretability and transparency
Fairness and accessibility											
Human–machine collaboration and humanistic care	0.685										
Overall acceptance	0.052	0.021									
Overall attitude	0.079	0.041	0.630								
Overall perceived risk	0.777	0.778	0.051	0.024							
Overall trust	0.126	0.132	0.624	0.575	0.174						
Personal trust propensity	0.127	0.124	0.468	0.554	0.140	0.500					
Privacy and data protection	0.680	0.652	0.263	0.075	0.744	0.047	0.188				
Responsibility and accountability	0.669	0.684	0.030	0.042	0.753	0.159	0.152	0.665			
Safety and reliability	0.675	0.661	0.026	0.024	0.711	0.131	0.098	0.644	0.651		
Technical interpretability and transparency	0.755	0.712	0.056	0.064	0.790	0.159	0.127	0.697	0.707	0.682	

Finally, multicollinearity diagnostics were conducted before structural model estimation. All variance inflation factor (VIF) values were below the conservative threshold of 3.3, indicating that multicollinearity was unlikely to bias the structural relationships. Detailed VIF results are reported in Supplementary Table S2.

### 4.3 Structural equation modeling analysis

On the basis of the satisfactory measurement model results, structural equation modeling was conducted to examine the hypothesized relationships among ethical concerns, overall perceived risk, overall trust, overall attitude, and overall acceptance.

Before interpreting individual structural paths, the overall fit of the structural model was assessed. The results indicated satisfactory model fit (*χ*^2^ = 1526.227, *df* = 912, *p* < 0.001, *χ*^2^/*df* = 1.673, GFI = 0.909, CFI = 0.979, TLI = 0.978, NFI = 0.950, RMSEA = 0.031), suggesting that the proposed structural relationships adequately represented the observed covariance structure.

The six ethical concern dimensions were simultaneously included as predictors of overall perceived risk to examine their unique conditional associations after accounting for shared variance among dimensions. As shown in Table 10. Privacy and data protection (*β* = 0.151), responsibility and accountability (*β* = 0.173), fairness and accessibility (*β* = 0.196), safety and reliability (*β* = 0.085), human–machine collaboration and humanistic care (*β* = 0.238), and technical interpretability and transparency (*β* = 0.187) all show significant positive associations with overall perceived risk, indicating that greater concern about these ethical issues is associated with higher overall perceived risk of medical AI. Therefore, H1a–H1f are all supported. Among them, human–machine collaboration and humanistic care has the strongest association, followed by fairness and accessibility and technical interpretability and transparency, indicating that the public’s risk judgment of medical AI is more concentrated on ethical aspects such as humanistic care, fairness, and transparency.

**Table 10:** Structural model path coefficients.

Hypothesis	Path	Standardized path coefficient *β*	C.R.	*p*-value
H1a	Privacy and data protection → overall perceived risk	0.151	4.282	***
H1b	Responsibility and accountability → overall perceived risk	0.173	4.877	***
H1c	Fairness and accessibility → overall perceived risk	0.196	5.043	***
H1d	Safety and reliability → overall perceived risk	0.085	2.498	0.012
H1e	Human–machine collaboration and humanistic care → overall perceived risk	0.238	6.477	***
H1f	Technical interpretability and transparency → overall perceived risk	0.187	4.568	***
H2	Overall perceived risk → overall trust	−0.240	−6.700	***
H3	Overall perceived risk → overall attitude	0.112	3.219	0.001
H4	Overall trust → overall attitude	0.608	15.275	***
H5	Overall trust → overall acceptance	0.395	9.394	***
H6	Overall attitude → overall acceptance	0.397	9.419	***
H7	Personal trust propensity → overall trust	0.555	13.265	***

*β* is the standardized path coefficient; ***Indicates *p* < 0.001.

Regarding the relationships among risk perception, trust, attitude, and acceptance, overall perceived risk is significantly negatively associated with overall trust (*β* = −0.240), indicating that higher risk perception is associated with lower public’s overall trust in medical AI, and H2 is supported. Overall perceived risk shows a significant positive association with overall attitude (*β* = 0.112), which does not support the hypothesized direction, therefore H3 is not supported. Given that the zero-order correlation between perceived risk and attitude was close to zero (*r* = −0.008, *p* > 0.05), this unexpected association should be interpreted cautiously. Overall trust shows significant positive associations with overall attitude (*β* = 0.608) and overall acceptance (*β* = 0.395), indicating that trust is associated with more positive attitudes and higher acceptance, and H4 and H5 are supported. Overall attitude shows a significant positive association with overall acceptance (*β* = 0.397), indicating that more positive evaluations of medical AI are associated with higher acceptance; therefore, H6 is supported.

In addition, personal trust propensity shows a significant positive association with overall trust (*β* = 0.555), indicating that a higher general trust tendency is associated with greater trust in medical AI; therefore, H7 is supported. Overall, the structural model results provide partial support for the proposed framework and reveal complex associations among ethical concerns, risk perception, trust, attitude, and acceptance.

All structural paths reported in Table 10 were estimated without control variables to reflect the theoretical model, while Table 11 presents a robustness check including demographic covariates (gender, age, education level, and prior experience with medical AI). The results indicate that the inclusion of these control variables does not substantially change the estimated path coefficients.

**Table 11:** Sensitivity analysis.

Path	Original model *β*	Control variable model *β*	*p*-value (control variable model)
Privacy and data protection → overall perceived risk	0.151	0.151	<0.001
Responsibility and accountability → overall perceived risk	0.173	0.173	<0.001
Fairness and accessibility → overall perceived risk	0.196	0.196	<0.001
Safety and reliability → overall perceived risk	0.085	0.085	0.012
Human–machine collaboration and humanistic care → overall perceived risk	0.238	0.238	<0.001
Technical interpretability and transparency → overall perceived risk	0.187	0.187	<0.001
Personal trust propensity → overall trust	0.555	0.554	<0.001
Overall perceived risk → Overall trust	−0.240	−0.240	<0.001
Overall perceived risk → overall attitude	0.112	0.111	0.001
Overall trust → overall attitude	0.608	0.608	<0.001
Overall trust → overall acceptance	0.395	0.406	<0.001
Overall attitude → overall acceptance	0.397	0.378	<0.001
Gender → overall acceptance	—	0.049	0.096
Age → overall acceptance	—	−0.016	0.586
Highest education → overall acceptance	—	−0.057	0.057
Used medical AI → overall acceptance	—	−0.045	0.130

**Figure 3:**
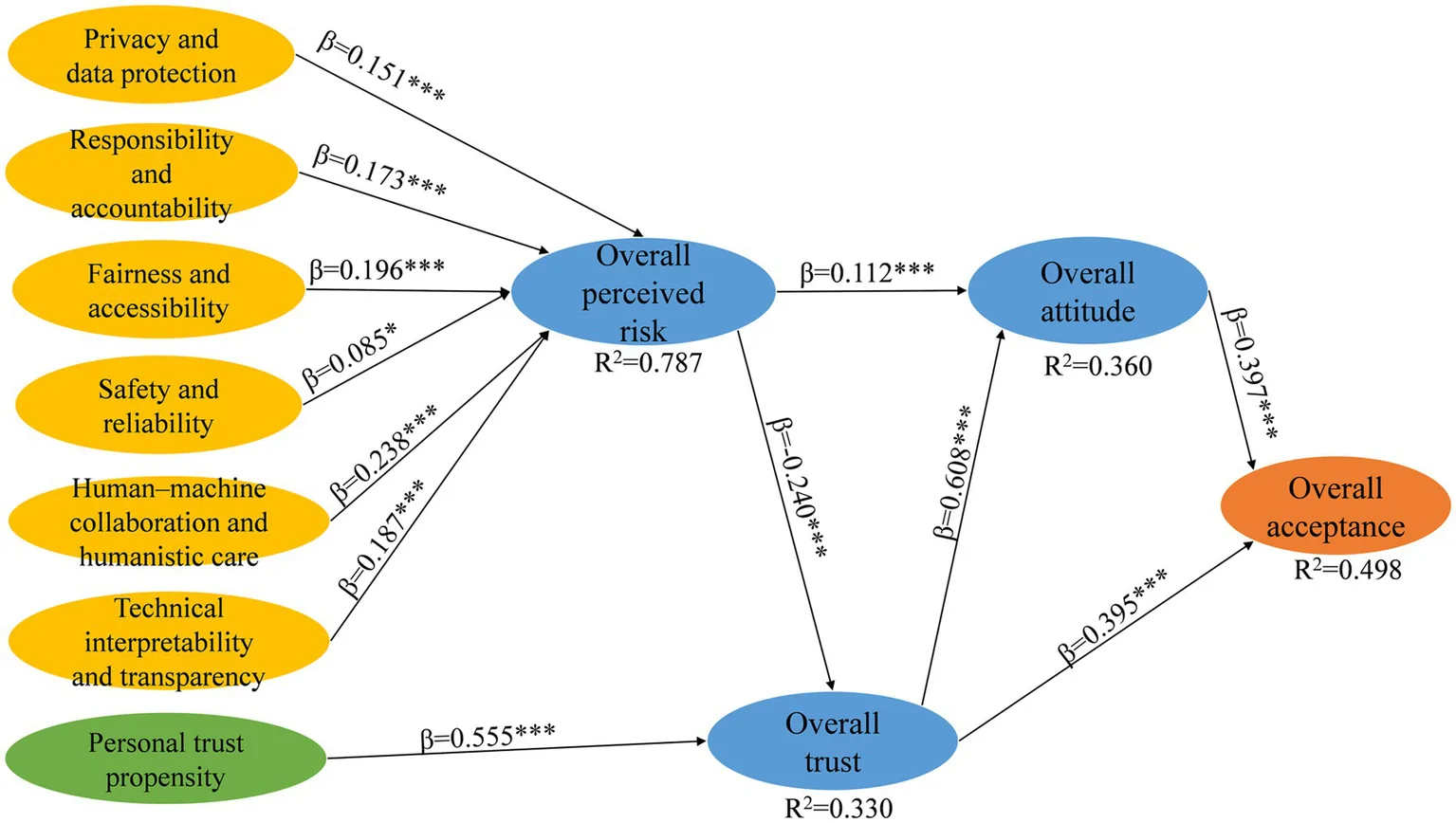
Standardized path coefficient results of the structural model. *, **, and ***Indicate significance at the 0.05, 0.01, and 0.001 levels, respectively; *R*^2^ represents the explained variance of the endogenous variables.

To more intuitively show the path relationships and standardized coefficients among latent variables in the structural model, this study further draws a standardized path result diagram of the structural model, as shown in Figure 3.

### 4.4 Indirect effect analysis of overall perceived risk, overall trust, and acceptance

To examine the indirect pathway specified in the structural model, bootstrap analysis with 5,000 resamples and bias-corrected confidence intervals was conducted based on the final structural model reported in Table 10. The results are presented in Table 12.

**Table 12:** Indirect effect analysis of overall perceived risk, overall trust, and acceptance.

Path	Indirect effect value	95% CI lower bound (BC)	95% CI upper bound (BC)
Overall perceived risk → overall trust → overall acceptance	−0.094	−0.156	−0.037

Bootstrap resampling times = 5,000; bias-corrected confidence intervals (BC) are used; indirect effect is significant when the 95% confidence interval does not include 0.

The results indicate that overall perceived risk is indirectly associated with overall acceptance through overall trust (indirect effect = −0.094, 95% BC CI excluding zero). This finding suggests that overall trust represents an indirect statistical pathway linking perceived risk and acceptance within the structural model.

However, the zero-order correlation between overall perceived risk and overall acceptance was not significant (*r* = 0.048, *p* > 0.05). Therefore, this indirect pathway should be interpreted as a statistical indirect association rather than evidence of causal mediation. The observed relationship reflects the interconnected associations among risk perception, trust, and acceptance within the proposed SEM framework.

### 4.5 Sensitivity analysis

To further exclude potential confounding by demographic characteristics, this study included gender, age, highest education level, and prior experience with medical AI as covariates in the structural model, specified to predict overall acceptance and freely correlated. The model parameters were re-estimated, and the results are shown in Table 11.

The re-estimation showed that after including the control variables, the direction and significance of all core paths remained essentially unchanged. Specifically, the effects of the six ethical concerns on overall perceived risk, the effect of overall perceived risk on overall trust, the effects of overall trust on overall attitude and overall acceptance, and the effect of overall attitude on overall acceptance all remained stable. This indicates that the findings are not driven by demographic differences. The covariates themselves had generally weak effects on overall acceptance, with most paths non-significant, implying that acceptance is primarily explained by the core psychological mechanisms.

It should be noted that this analysis is not intended to serve as a formal test of cross-group robustness; such a test would require measurement invariance and multi-group structural equation modeling, which remain an avenue for future research.

## 5 Discussion

### 5.1 General attitudes toward medical AI

The descriptive statistics show that mean scores for overall acceptance (M = 5.136), overall trust (M = 5.730), and overall attitude (M = 5.171) are above the midpoint of the 7-point scale, indicating that respondents in this sample generally held favorable evaluations of medical AI despite the presence of ethical concerns and perceived risks.

Comparison between respondents with and without prior exposure to medical AI shows that participants with prior experience reported slightly higher perceived risk scores (M = 4.122, SD = 1.220) than those without prior exposure (M = 3.912, SD = 1.530). Because the homogeneity-of-variance assumption was not met, Welch’s independent-samples t-test was used. The difference was statistically significant but small, *t*(684.28) = −2.02, *p* = 0.044, Cohen’s d = 0.15. Given the small effect size and the broad, dichotomous measurement of prior exposure, this result should be interpreted cautiously and regarded as exploratory. Future research should examine whether, and under what conditions, prior exposure is associated with perceived risk toward medical AI. More broadly, evaluations of medical AI among the surveyed participants appear to involve multiple interconnected cognitive and ethical considerations rather than a single perceptual dimension.

### 5.2 Perceptions of ethical concerns in medical AI

Concerns across the six ethical dimensions were not evenly distributed among respondents. Responsibility and accountability and safety and reliability received relatively higher concern scores, followed by human–machine collaboration and humanistic care (Figure 3). These patterns suggest that respondents in this sample were particularly attentive to issues related to institutional responsibility, system dependability, and the preservation of human-centered care in medical AI applications.

Beyond these descriptive differences, the second-order CFA further indicates that the six ethical dimensions collectively represent an overarching ethical concern construct. Meanwhile, the structural model demonstrates that different ethical dimensions vary in their associations with overall perceived risk. Specifically, human–machine collaboration and humanistic care, fairness and accessibility, and technical interpretability and transparency showed relatively stronger associations with perceived risk. This finding suggests that concerns regarding human involvement, fairness, and transparency may be particularly relevant in shaping risk perceptions of medical AI.

From a theoretical perspective, Maslow’s hierarchy of needs has been discussed as a framework for understanding the importance of safety and interpersonal values in human evaluations of emerging technologies (24). Similarly, intolerance of uncertainty provides a useful perspective for considering concerns related to system interpretability and transparency. These perspectives are used here as interpretative tools rather than causal explanations. Together, they highlight that ethical concern toward medical AI involves multiple layers of evaluation, including technical, social, and relational considerations.

### 5.3 Associations among risk perception, trust, attitude, and acceptance

Building on the hierarchical ethical concern framework, the results show that perceived risk is negatively associated with trust, while trust is positively associated with both attitude and acceptance. Attitude is also positively associated with acceptance. These findings indicate that trust is an important construct associated with the relationships among risk perception, attitude, and acceptance. However, given the cross-sectional design, these relationships should be interpreted as statistical associations rather than causal mechanisms.

Higher perceived risk was not necessarily associated with lower acceptance among surveyed participants. Instead, the positive association between perceived risk and attitude suggests that risk awareness and favorable evaluations of medical AI may coexist under certain conditions. This unexpected association warrants further investigation, as it may reflect complex evaluation processes in which individuals simultaneously consider potential risks and potential benefits of medical AI.

### 5.4 Risk perception and positive attitudes toward medical AI: a possible risk–benefit trade-off perspective

An unexpected finding of this study is that perceived risk was positively associated with attitude toward medical AI, which differs from the originally hypothesized negative relationship. Although this association should be interpreted cautiously due to the complexity of the structural relationships among closely related constructs, it may reflect a possible risk–benefit trade-off in individual evaluations of medical AI.

Unlike general consumer technologies, medical AI is directly related to healthcare quality and clinical decision-making, where potential benefits may be considered valuable despite the existence of risks. Respondents may simultaneously recognize potential concerns related to privacy, accountability, and system reliability while acknowledging the potential benefits of medical AI in improving healthcare efficiency and clinical decision support. Therefore, higher perceived risk does not necessarily indicate lower acceptance of medical AI; instead, risk awareness may coexist with favorable evaluations when individuals consider both potential risks and potential benefits. Future research may further examine whether factors such as technological literacy, prior experience with medical AI, or other individual characteristics influence this relationship.

### 5.5 Indirect association among risk, trust, and acceptance

The indirect association analysis indicates that perceived risk is indirectly associated with acceptance through trust. This suggests that trust represents an important statistical pathway linking perceived risk and acceptance within the structural model. Given the non-significant zero-order association between perceived risk and acceptance, this indirect pathway should be interpreted as an indirect statistical association rather than a causal mediation mechanism. These findings indicate that acceptance of medical AI among surveyed participants involves interconnected evaluations of risk perception and trust rather than a single linear decision process.

## 6 Implications

### 6.1 Risk governance and system safety

Perceived risk appears closely related to how surveyed participants evaluate the reliability and controllability of medical AI systems. This suggests that risk perception is shaped not only by technical performance but also by perceived uncertainty in system behavior. Improvements in transparency and reliability may be associated with more favorable evaluations of medical AI among surveyed participants. Nevertheless, these relationships should be interpreted as associative rather than deterministic.

### 6.2 Privacy and user autonomy

Privacy and data protection represent an important ethical dimension in the evaluation of medical AI among surveyed participants. These concerns may reflect both the sensitivity of medical data and users’ perceived control over personal information. Greater clarity in data usage and consent processes may be associated with improved perceptions of autonomy and security. However, the strength of these associations requires further empirical validation. Although privacy represents a specific ethical domain, it should be understood as part of a broader ethical concern structure rather than an isolated issue.

### 6.3 Fairness and accountability

Concerns regarding fairness, accessibility, responsibility, and accountability reflect expectations for equitable healthcare access and transparent responsibility allocation. These concerns appear to be closely tied to broader perceptions of fairness in algorithmic decision-making and institutional governance structures. Improvements in these areas may correspond with more favorable evaluations of medical AI.

### 6.4 Human–machine collaboration and humanistic care

Human–machine collaboration and humanistic care represents an important dimension of ethical concern and shows a relatively strong association with perceived risk in this study. This finding suggests that evaluations among surveyed participants are associated not only with technical performance but also with whether AI is perceived as supporting clinicians and maintaining human-centered care.

Therefore, medical AI should be positioned as a supportive tool that enhances rather than replaces human decision-making. Enhancing human–AI collaboration and preserving meaningful clinician–patient interactions may be associated with more favorable evaluations of medical AI among surveyed participants. Importantly, this dimension should be understood as a specific manifestation of the broader ethical concern construct rather than an isolated ethical issue.

## 7 Conclusion and future directions

### 7.1 Conclusion

This study identifies and validates a hierarchical multidimensional structure of ethical concern in medical AI (25). All six dimensions—privacy and data protection, fairness and accessibility, responsibility and accountability, safety and reliability, human–machine collaboration and humanistic care, and technical interpretability and transparency—are significantly associated with overall perceived risk (8). These dimensions collectively form a higher-order ethical concern construct while retaining their domain-specific characteristics. The results further suggest that overall trust is statistically involved in the association between overall perceived risk and overall acceptance (16). Overall, acceptance of medical AI among surveyed participants is associated with interconnected relationships among perceived risk, trust, and attitude rather than a single linear evaluation process.

### 7.2 Limitations and future research

Several limitations should be acknowledged when interpreting the findings of this study.

First, the sample included respondents across different age groups and educational backgrounds, although younger adults represented a relatively proportion of the sample. In addition, 41.32% of participants reported prior experience with medical AI. These sampling characteristics may limit the generalizability of the findings to broader populations. Future studies should consider more diverse sampling strategies, including respondents with different demographic, socioeconomic, healthcare-related, and technology-exposure backgrounds.

Second, although the second-order CFA provided support for representing the six ethical concern dimensions as a higher-order construct, the relative salience and interpretation of specific ethical dimensions may vary across cultural contexts and medical AI applications. Future research should further validate this hierarchical structure using samples from different regions and application scenarios.

Third, although the measurement model demonstrated satisfactory validity, some ethical concerns may overlap with general emotional responses toward emerging technologies. Future research could further refine measurement approaches to distinguish ethical evaluations, perceived risks, and affective reactions toward medical AI.

Fourth, the cross-sectional design limits causal interpretation of the relationships among ethical concerns, perceived risk, trust, attitude, and acceptance. In particular, the unexpected positive association between perceived risk and attitude should be interpreted cautiously and does not necessarily indicate a causal psychological mechanism. Longitudinal or experimental studies are needed to further examine the temporal relationships and potential boundary conditions underlying these associations.

Finally, although potential common method variance (CMV) was assessed using Harman’s single-factor test and the Common Latent Factor approach, all variables were collected through self-reported questionnaires at a single time point. Therefore, the potential influence of common method-related effects cannot be completely excluded. Although the CLF analysis suggested that CMV was unlikely to account for the majority of variance in the observed relationships, self-reported measures may still introduce certain response biases. Future studies may incorporate multi-source data, longitudinal designs, behavioral indicators, or objective measures to further reduce potential common method effects and strengthen the robustness of the findings.

## Data Availability

The datasets presented in this article are not readily available because the dataset is not publicly available due to ethical restrictions related to participant privacy and data protection. The data were collected under an approved ethics protocol that did not include authorization for unrestricted public data sharing. Fully anonymized datasets may be available from the corresponding author upon reasonable request and subject to appropriate review of the intended use. Requests to access the datasets should be directed to joey@ntu.edu.cn.
