# Supplementary material for: Ethical concerns toward medical artificial intelligence and acceptance intentions: a structural equation modeling analysis of the risk perception–trust pathway
Source: Front Public Health. 2026 Aug 25;14:1858239. doi: 10.3389/fpubh.2026.1858239 (PMC13550962; doi:10.3389/fpubh.2026.1858239)
Supplement: Supplementary file 1 [file Supplementary_file_1.pdf]

Supplementary Materials

Table S1 Factor Loading Coefficients and Comprehensive Model Evaluation

Variable	Standardized Loading Coefficient	AVE Value	CR Value
Personal trust propensity (adapted from Mayer and Davis, 1999; based on Rotter, 1967[28,29])		0.656	0.884
I am willing to give initial trust when building relationships with others.	0.782		
I think most people are trustworthy in cooperation.	0.855		
I tend to trust others first even without evidence.	0.784		
I am usually willing to believe that others are honest and reliable.	0.822		
Privacy and data protection		0.786	0.936
I will be cautious about AI applications without independent third-party privacy reviews.	0.905		
I feel more assured to use medical AI only if it clearly explains how data is protected.	0.909		
I worry that data and information related to medical AI will be misused in sharing or cooperation.	0.867		
I worry that medical AI will leak my personal health data.	0.857		
Responsibility and accountability		0.797	0.940
I hope hospitals formulate internal accountability procedures to clarify internal investigation and accountability processes when medical AI makes mistakes.	0.897		

I support establishing more complete legal regulations for AI medical treatment.	0.910		
I hope to be clearly informed who is responsible for errors before using AI.	0.851		
I worry about unclear responsibility attribution when medical AI makes mistakes (e.g., doctors, hospitals, or manufacturers).	0.910		
Fairness and accessibility		0.764	0.928
Improving efficiency should not come at the cost of sacrificing medical access opportunities for vulnerable groups.	0.878		
I think fairness assessment results of medical AI across different genders, ages, and regions should be regularly disclosed.	0.890		
Medical AI promotion should avoid excluding groups with weak intelligent device capabilities.	0.880		
I worry that medical AI mainly learns data from healthy young people, and the elderly or special patients may not receive fair services.	0.846		
Safety and reliability		0.784	0.936
I worry that system crashes or interruptions of medical AI will directly threaten patient safety.	0.874		
I think medical AI needs regular safety assessments by independent institutions.	0.870		
Medical AI should be equipped with real-time error monitoring and trigger manual review.	0.909		
I worry that medical AI may make misjudgements in complex or emergency situations.	0.889		

Human–machine collaboration and humanistic care		0.759	0.926
I worry that personalized care for patients will decrease after doctors rely on AI.	0.878		
I worry that the "efficiency first" orientation will marginalize humanistic care.	0.872		
I worry that doctors will reduce communication and comfort with patients after using AI.	0.885		
I worry that doctors' over-reliance on AI will lead to a decline in clinical judgment ability.	0.849		
Technical interpretability and transparency		0.821	0.948
Just as doctors explain test results, I hope AI can also explain its reasoning process.	0.904		
I hope medical AI has a diagnostic traceability function to trace the basis data.	0.908		
I hope to see key indicators or reasons to know how AI reaches conclusions.	0.907		
I find it hard to trust the results of medical AI if it cannot explain the diagnostic basis.	0.904		
Overall perceived risk (developed based on AIRBS and AI risk literature[30])		0.779	0.946
Even if medical AI is certified, I still think potential risks are difficult to completely eliminate.	0.886		
I worry that the risks of medical AI lack effective supervision and constraints.	0.881		
I think the risks of medical AI are harder to detect and correct than traditional medical treatment.	0.882		
I worry that medical AI may cause serious consequences (e.g., misdiagnosis, delay).	0.887		

Overall, I worry about uncertainties in the application process of medical AI.	0.877		
Overall attitude (adapted from AIAS-4; based on UTAUT framework[31,3])		0.721	0.912
I support the standardized use of medical AI.	0.850		
I am optimistic about the future development of medical AI.	0.840		
I believe medical AI can improve medical experience and efficiency.	0.848		
I think medical AI is generally worth promoting.	0.858		
Overall trust (adapted from McKnight et al., 2002; based on Mayer et al. trust framework[32,33])		0.730	0.915
I believe medical AI can provide stable support in most common disease scenarios.	0.858		
I think medical AI is reliable in most cases.	0.869		
I will trust the diagnostic results of medical AI if fully verified.	0.852		
On the recommendation of a doctor, I am willing to refer to the diagnosis or treatment suggestions of medical AI.	0.838		
Overall acceptance (adapted from Davis, 1989; Venkatesh et al., 2003[2,3])		0.746	0.922
Under the guidance of doctors, I am willing to let medical AI participate in treatment as an auxiliary tool.	0.858		
On the recommendation of a doctor, I am willing to try diagnosis and treatment services involving medical AI.	0.869		

If the experience is good, I will recommend family and friends to use it.	0.892
I am willing to use medical AI-related services in the future.	0.838

Note: The six ethical concern constructs (privacy and data protection, responsibility and accountability, fairness and accessibility, safety and reliability, human–machine collaboration and humanistic care, and technical interpretability and transparency) were developed based on prior literature in AI ethics and risk perception, and were refined to fit the medical AI context. These constructs were not adapted from a single existing measurement scale.

Table S2 Multicollinearity Test

Dependent Variable	Independent Variable	VIF
Overall trust	Personal trust	1.013
Overall trust	Overall perceived risk	1.013
Overall perceived risk	Privacy and data protection	2.217
Overall perceived risk	Responsibility and accountability	2.302
Overall perceived risk	Fairness and accessibility	2.490
Overall perceived risk	Safety and reliability	2.157
Overall perceived risk	Human–machine collaboration and humanistic care	2.293
Overall perceived risk	Technical interpretability and transparency	2.814
Overall attitude	Overall trust	1.027
Overall attitude	Overall perceived risk	1.027
Overall acceptance	Overall attitude	1.380
Overall acceptance	Overall trust	1.380

Note: VIF values were calculated using construct mean scores as observed indicators for latent variables. Each endogenous construct was examined according to its corresponding structural equation in the SEM model.

Table S3 Common Latent Factor (CLF) Test Results

**Item**	**Construct**	**CFA Loading**	**CLF Loading**	**△ (CFA -CLF)**
x7	Privacy and data protection	0.905	0.344	0.561

x6	Privacy and data protection	0.909	0.349	0.56
x5	Privacy and data protection	0.867	0.372	0.495
x4	Privacy and data protection	0.857	0.364	0.493
x11	Responsibility and accountability	0.897	0.368	0.529
x10	Responsibility and accountability	0.91	0.358	0.552
x9	Responsibility and accountability	0.91	0.368	0.542
x8	Responsibility and accountability	0.851	0.376	0.475
x15	Fairness and accessibility	0.878	0.357	0.521
x14	Fairness and accessibility	0.89	0.356	0.534
x13	Fairness and accessibility	0.88	0.361	0.519
x12	Fairness and accessibility	0.846	0.373	0.473
x19	Safety and reliability	0.874	0.364	0.51
x18	Safety and reliability	0.87	0.363	0.507
x17	Safety and reliability	0.909	0.36	0.549
x16	Safety and	0.889	0.369	0.52

	reliability			
x23	Human–machine collaboration and humanistic care	0.878	0.374	0.504
x22	Human–machine collaboration and humanistic care	0.872	0.381	0.491
x21	Human–machine collaboration and humanistic care	0.885	0.378	0.507
x20	Human–machine collaboration and humanistic care	0.849	0.374	0.475
x27	Technical interpretability and transparency	0.904	0.336	0.568
x26	Technical interpretability and transparency	0.907	0.341	0.566
x25	Technical interpretability and transparency	0.908	0.346	0.562

x24	Technical interpretability and transparency	0.904	0.348	0.556
x3	Personal Trust propensity	0.782	0.439	0.343
x2	Personal Trust propensity	0.855	0.442	0.413
x1	Personal Trust propensity	0.784	0.392	0.392
x0	Personal Trust propensity	0.822	0.427	0.395
x28	Overall perceived risk	0.877	0.381	0.496
x29	Overall perceived risk	0.887	0.375	0.512
x30	Overall perceived risk	0.882	0.383	0.499
x31	Overall perceived risk	0.881	0.371	0.51
x32	Overall perceived risk	0.886	0.373	0.513
x33	Overall attitude	0.858	0.444	0.414
x34	Overall attitude	0.848	0.453	0.395
x35	Overall attitude	0.84	0.458	0.382
x36	Overall attitude	0.85	0.444	0.406
x40	Overall trust	0.858	0.485	0.373
x39	Overall trust	0.869	0.486	0.383
x38	Overall trust	0.852	0.504	0.348

x37	Overall trust	0.838	0.503	0.335
x41	Overall acceptance	0.838	0.435	0.403
x42	Overall acceptance	0.892	0.447	0.445
x43	Overall acceptance	0.869	0.44	0.429
x44	Overall acceptance	0.858	0.432	0.426

Note: CFA = standardized factor loadings from the confirmatory factor analysis model; CLF = standardized loadings after introducing a common latent factor; Δ = difference between CFA and CLF loadings.

Table S4 Comparison of perceived risk between respondents with and without prior medical AI exposure

Used medical AI	N	Mean	SD	Welch's t	df	p-value	Cohen's d
No	409	3.912	1.530	-2.019	684.275	0.044	0.149
Yes	288	4.122	1.220				
